# Incidental Hydroxyapatite Ocular Implant Uptake on Bone Scan Done for Prostate Cancer Staging: Case Report and Brief Review

**DOI:** 10.4274/mirt.galenos.2019.52386

**Published:** 2019-06-24

**Authors:** Guillaume Chaussé, Jerome Laufer, Gad Abikhzer, Stephan Probst

**Affiliations:** 1 McGill University Faculty of Medicine, Department of Radiology, Division of Nuclear Medicine, Montreal, Canada

**Keywords:** Tc-99m MDP, bone scan, ocular implant, artificial eye, eye prosthesis, hydroxyapatite

## Abstract

A 74-year-old man recently diagnosed with high-risk prostate cancer with high serum prostate specific antigen was referred to nuclear medicine for a technetium-99m-methylene diphosphonate (Tc-99m MDP) bone scan. On delayed three-hour anterior planar image, an unexpected round focus of intense uptake was found overlying the right orbit. Single-photon emission computed tomography/computed tomography localized the uptake to an ocular prosthesis. The hydroxyapatite composition of the ocular implant can be recognized by its bone-like density and its intense accumulation of Tc-99m MDP. Review of the patient’s history revealed remote right eye evisceration secondary to a complication of cataract surgery, consistent with the findings.

## Abstract (tr)

Yakın zamanda yüksek riskli prostat kanseri tanısı almış, serum prostat spesifik antijen seviyesi yüksek 74 yaşında bir erkek hasta teknesyum-99m-metilen difosfonat (Tc-99m MDP) kemik sintigrafisi için nükleer tıp bölümüne yönlendirildi. Gecikmiş üç saatlik anterior planar görüntüde sağ orbita üzerinde beklenmeyen yuvarlak ve yoğun tutulum odağı saptandı. Tek-foton emisyon bilgisayarlı tomografi/bilgisayarlı tomografi tutulumu oküler protezde lokalize etti. Oküler implantın hidroksiapatit bileşeni kemiğe benzer dansite ve yoğun Tc-99m MDP tutulumu ile tanınabilir. Hastanın özgeçmişinde, bulgularla uyumlu şekilde, katarakt cerrahisi komplikasyonuna sekonder sağ göz eviserasyonu saptandı.

## Figures and Tables

**Figure 1:**
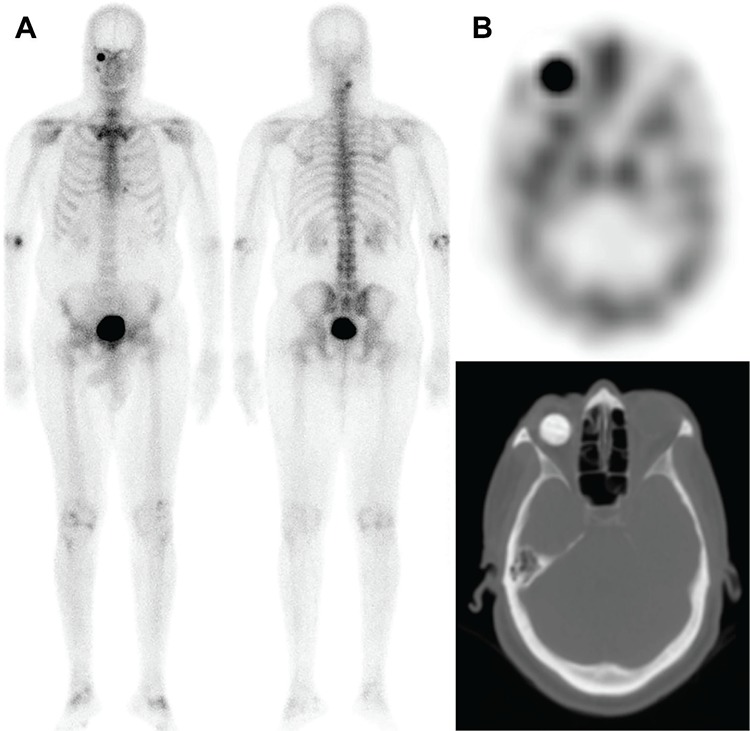
A 74-year-old man recently diagnosed with high-risk prostate cancer with high serum prostate specific antigen was referred to nuclear medicine for a technetium-99m-methylene diphosphonate (Tc-99m MDP) bone scan. As per institutional protocol, whole-body blood pool images were acquired, which were unremarkable (not shown). A) On delayed three-hour planar images, an unexpected round focus of intense uptake was found overlying the right orbit. B) Selected axial slice of Tc-99m MDP single-photon emission computed tomography/computed tomography showing intense uptake throughout a right hydroxyapatite ocular prosthesis. The hydroxyapatite nature of the ocular implant can be recognized by its bone-like density and its accumulation of bone scan agent. Review of the patient’s history revealed right eye evisceration 20 years prior secondary to a complication of cataract surgery, consistent with the findings. Although they are more costly, hydroxyapatite ocular implants offer many advantages over non-integrated implants. Thanks to their porous surface and organic composition, they allow in-growth of tissue, are lighter and allow insertion of a peg-a small pin-like device that improves coupling of the eyeball to the overlying artificial eye (1). A painful blind eye, cosmetics or trauma are reasons for their use. Evisceration, a process by which the inner content of the eyeball is removed by preserving the sclera, is then followed by insertion of the hydroxyapatite implant. Fibrovascular in-growth provides minimal risk of rejection, infection or migration (1). Radionuclide bone scan has been used to assess vascularization of eye prosthesis, an essential prerequisite prior to the drilling of the peg hole. Civelek et al. (2) demonstrated that semi-quantitative measurement by means of implanted to non-implanted eye ratios of uptake on bone scan identifies proper vascularization with high specificity. However, it seems that distribution of activity throughout the whole implant, rather than simply the intensity of the uptake, predicts greater likelihood of success (2,3). The peg hole must be conjunctivized, and therefore requires complete vascularization of the implant before being drilled. As uptake on bone scan is proportional to the vascularization of the implant, it can be used to assess the timing of complete vascularization, which usually occurs at around four months postimplantation (4). Counterintuitively, bone scan early phase “flow” studies and blood pool image analysis are not useful in this regard, and only delayedphase imaging reliably correlates with vascularization; it is hypothesized that fibrovascular tissue lacks sizeable arteries to be detected by such means (5). Our case illustrates interesting but normal incidental ocular prosthetic uptake which is infrequently seen. Bone scan can be used to guide early implant management, but this finding can be encountered 20 or more years after hydroxyapatite ocular implant insertion
